# OrthoClusterDB: an online platform for synteny blocks

**DOI:** 10.1186/1471-2105-10-192

**Published:** 2009-06-23

**Authors:** Man-Ping Ng, Ismael A Vergara, Christian Frech, Qingkang Chen, Xinghuo Zeng, Jian Pei, Nansheng Chen

**Affiliations:** 1 Department of Molecular Biology and Biochemistry, Simon Fraser University, 8888 University Drive, Burnaby, V5A 1S6, Canada; 2 School of Computing Science, Simon Fraser University, 8888 University Drive, Burnaby, V5A 1S6, Canada

## Abstract

**Background:**

The recent availability of an expanding collection of genome sequences driven by technological advances has facilitated comparative genomics and in particular the identification of synteny among multiple genomes. However, the development of effective and easy-to-use methods for identifying such conserved gene clusters among multiple genomes–synteny blocks–as well as databases, which host synteny blocks from various groups of species (especially eukaryotes) and also allow users to run synteny-identification programs, lags behind.

**Descriptions:**

OrthoClusterDB is a new online platform for the identification and visualization of synteny blocks. OrthoClusterDB consists of two key web pages: *Run OrthoCluster *and *View Synteny*. The *Run OrthoCluster *page serves as web front-end to OrthoCluster, a recently developed program for synteny block detection. *Run OrthoCluster *offers full control over the functionalities of OrthoCluster, such as specifying synteny block size, considering order and strandedness of genes within synteny blocks, including or excluding nested synteny blocks, handling one-to-many orthologous relationships, and comparing multiple genomes. In contrast, the *View Synteny *page gives access to perfect and imperfect synteny blocks precomputed for a large number of genomes, without the need for users to retrieve and format input data. Additionally, genes are cross-linked with public databases for effective browsing. For both *Run OrthoCluster *and *View Synteny*, identified synteny blocks can be browsed at the whole genome, chromosome, and individual gene level. OrthoClusterDB is freely accessible.

**Conclusion:**

We have developed an online system for the identification and visualization of synteny blocks among multiple genomes. The system is freely available at .

## Background

Accumulating evidence suggests that genes within a genome are not randomly distributed. Instead, they form various types of conserved gene clusters, such as operons [1,2], genes co-regulated by common transcription mechanisms [3], and genes co-expressed in a same tissue type such as muscle [4]. The recent availability of an expanding collection of genome sequences driven by technological advances has facilitated genome-wide detection of these functional gene clusters through comparative genome analysis [5]. However, the development of effective and easy-to-use methods for identifying such conserved gene clusters among multiple genomes–synteny blocks–that at the same time host databases of these synteny blocks lags behind.

The term synteny has been used to refer different concepts in the past. Initially, synteny was used to indicate that genes are located on the same chromosome [6]. Recently, synteny has been more generally used to describe conservation, and syntenic genes have been generally taken as genes co-localized within conserved genomic blocks among related genomes [7]. There are further differences regarding the level of conservation. For example, some define two genomic sequences as a synteny block as long as they contain orthologous gene sets regardless of their order [8] or the existence of insertion/deletions [9]. In this paper, we generally follow recent definitions of synteny block and define it as a "genomic region of conserved gene content". We distinguish between "perfect synteny blocks" (genomic regions of perfectly conserved gene content, including gene order and strandedness) and "imperfect synteny blocks" (genomic regions of imperfectly conserved gene content, order or strandedness).

Most methods developed in the past years for detecting synteny blocks cannot be generally applied because they fail in one or more of the following tasks: (A) comparing multiple genomes; (B) detecting synteny blocks containing interruptions (mismatches); (C) considering strandedness (orientation) of genes; and (D) handling one-to-many orthologous relationships (reviewed in [10]). To overcome the above limitations, we have recently developed a program, OrthoCluster, for synteny block detection [10].

To make it easy for users to run OrthoCluster and to interpret the output, we have now developed a web server, OrthoClusterDB , which provides an easy-to-use web interface to OrthoCluster and immediate access to synteny blocks that have been precomputed with OrthoCluster. Currently, only two synteny detection methods–Cinteny [11] and SyMAP [12]–also provide servers for online access and access to databases.

## Construction and content

The OrthoClusterDB website consists of the following two key web pages: *Run OrthoCluster *and *View Synteny*. The *Run OrthoCluster *web page enables users to run OrthoCluster with their own genome annotation files and correspondence files (containing orthologous relationships among all input genomes) to identify synteny blocks. The *View Synteny *web page allows users to browse through pre-computed synteny blocks between up to three genomes at the genome, chromosome and gene level. In addition to these two pages, OrthoClusterDB also has a *Download *page, which provides users with the datasets used for generating the pre-computed results and OrthoCluster executables, and a *Help *page, which includes answers to frequently asked questions, protocols for using *Run OrthoCluster *and *View Synteny *pages, and the OrthoCluster tutorial.

## Utility and discussion

### Run OrthoCluster

**Figure 1:**
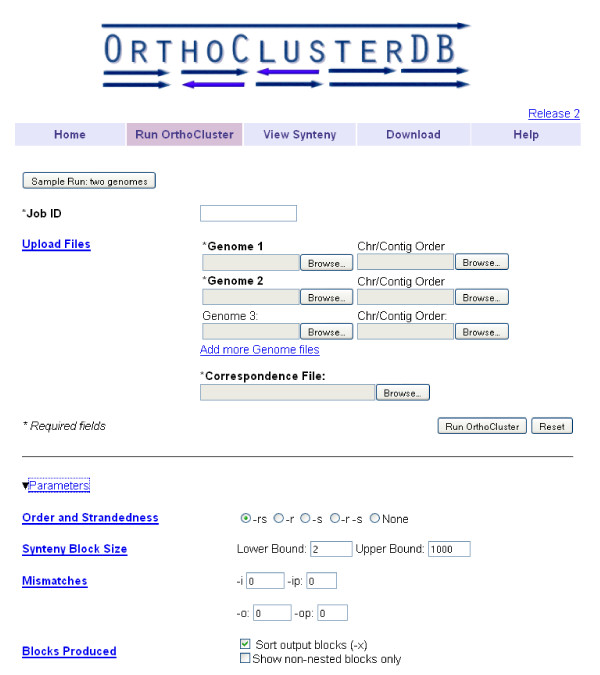
**Web interface of the *Run OrthoCluster *page showing the input parameters**.

The *Run OrthoCluster *page allows users to run OrthoCluster online using their own genome annotation and correspondence files to identify synteny blocks among a large number of genomes (Figure 1). Before running OrthoCluster online, users are recommended to check whether their genomes of interest are already included in the precomputed datasets in the *View Synteny *page. We provide details regarding where genome annotations are obtained together with release version number for each genome so that users can track down the data source for accurate analysis and comparison.

Two types of files are needed as input, the *genome file *and the *correspondence file *(both are plain tab-delimited text files). A genome file contains all genes and their coordinates in an input genome, while the correspondence file contains orthologous relationships among all input genomes. Users can define and modify parameters for running OrthoCluster, such as block size, order and strandedness of genes within synteny blocks, and inclusion/exclusion of nested synteny blocks resulting from one-to-many orthologous relationships. Notably, even though most users run OrthoCluster using genes and their orthologous relationships as input, OrthoCluster can be used to process any type of genomic feature (or genetic markers) as long as their orthologous relationships are provided.

Users can upload two or more input genomes. The first input genome ("Genome 1") is by default taken as the *reference genome *and the rest are referred to as the *target genomes*. By default, perfect synteny blocks will be generated.

**Figure 2:**
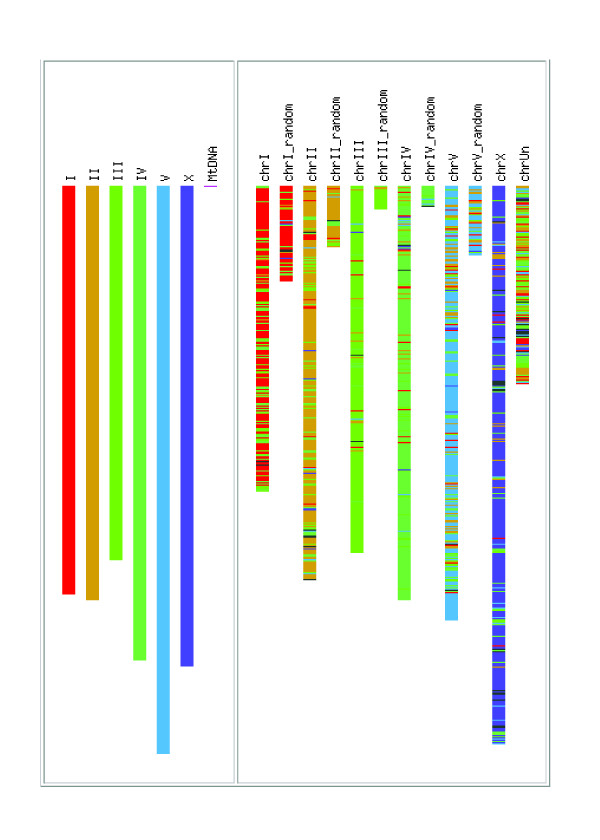
**A sample output *Genome Painter *image, with a link for output download and a link to GBrowse**. In this example, *C. elegans *is the reference genome and *C. briggsae *is the target genome.

The main part of the result page consists of the *Genome Painter *image that displays an overview of detected synteny blocks between a reference genome and target genome(s) at the chromosome/contig-level (Figure 2). This is achieved by first partitioning the reference genome into segments of different colors. (A) For reference genomes with 50 or less chromosomes/contigs but more than one chromosome/contig, each chromosome/contig gets assigned a different color and is shown in a separate column with its corresponding name. (B) For reference genomes with more than 50 chromosomes/contigs but fewer than 256 chromosomes/contigs, chromosomes/contigs are drawn with a continuous color gradient and without displaying their name for clarity. (C) For reference genomes containing more than 256 chromosomes/contigs, only the first 256 largest chromosomes/contigs are assigned unique colors and chromosomes/contigs beyond this number will be assigned the same color (black). (D) For reference genomes that are composed of only one chromosome/contig, the chromosome/contig is colored in a rainbow-spectrum manner. Detected synteny blocks are then highlighted within target genomes by drawing all syntenic regions in the color of their corresponding segment in the reference genome. The gray color in the target genomes indicates that no corresponding synteny blocks have been found in that region. By default, the order of the chromosomes/contigs displayed is sorted by size.

**Figure 3:**
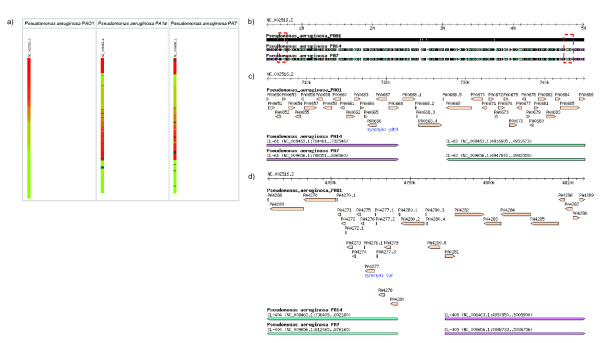
**Large inversion in *Pseudomonas aeruginosa *genomes**. a) Genome Painter image of three *P. aeruginosa *genomes, showing a large inversion of the two target genomes with respect to the reference genome (*P. aeruginosa *PAO1). b) GBrowse image of the large inverted region. The two junctions are surrounded by red dashed boxes. c) GBrowse image of the left-most junction of the inverted region. d) GBrowse image of the right-most junction of the inverted region.

The *Genome Painter *image is particularly useful for visualizing overall conservation of different genomes. For example, as illustrated in Figure 3, there is an obvious large inversion between the *Pseudomonas aeruginosa *PAO1 genome and the genomes of *Pseudomonas aeruginosa *PA14 and *Pseudomonas aeruginosa *PA7, as reported previously [13]. In contrast, the genomes of *Pseudomonas aeruginosa *PA14 and *Pseudomonas aeruginosa *PA7 are generally very similar.

**Figure 4:**
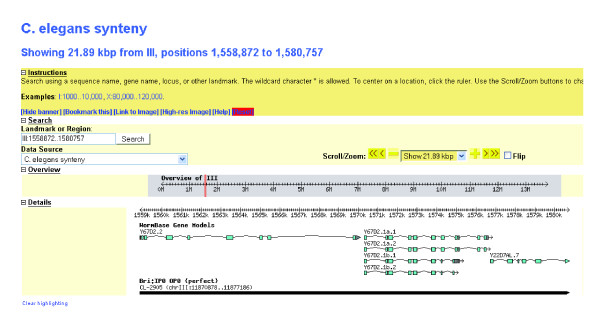
**GBrowse-based synteny browser with *C. elegans *as reference genome and *C. briggsae *as target genome**. The first track shows the WormBase gene model for *C. elegans*, and the second track is the synteny block detected in *C. briggsae*. CL-2905 is the synteny block ID assigned by OrthoCluster, and the number in brackets next to the ID refers to the chromosome location of the block in *C. briggsae*

Each synteny block within the *Genome Painter *image is clickable and cross-linked to a genome browser for gene-level view of the chromosomal/contig region containing that block. We use the Generic Genome Browser (GBrowse) [14] for that purpose, a widely used genome browser program (Figure 4). Users can enter the GBrowse view either by clicking on the link in the result summary table, or by directly clicking on the color-coded synteny blocks in the genome painter image. We configured GBrowse to display the genes of the reference genome in one track and the corresponding synteny blocks of the target genomes in separate tracks. Each synteny block displayed in the genome browser in the target genome is cross-linked to another genome browser, in which the target genome is displayed as the reference for that synteny block.

The *Run OrthoCluster *page also allows users to redefine the default behavior of OrthoCluster as well as the *Genome Painter *output. First, users can modify the order of chromosomes/contigs in the *Genome Painter *image by uploading a simple text file containing all the chromosome/contig names in a desired order. Format details can be found in the *Help *page. Second, users can generate various types of imperfect synteny blocks by varying the parameters of OrthoCluster, such as the minimum and maximum number of genes within the block, number/percentage of in-map mismatches (i.e. genes with known but non-syntenic orthologs) and out-map mismatches (genes without known orthologs) in a block, and preservation of the relative order and strandedness of orthologous genes within each synteny block. Additionally, the user is allowed to display non-nested synteny blocks only. Nested synteny blocks within larger blocks occur because of one-to-many orthologous relationships, which are usually present in the correspondence file and which OrthoCluster considers simultaneously at the moment of generating the synteny block.

### View synteny

**Figure 5:**
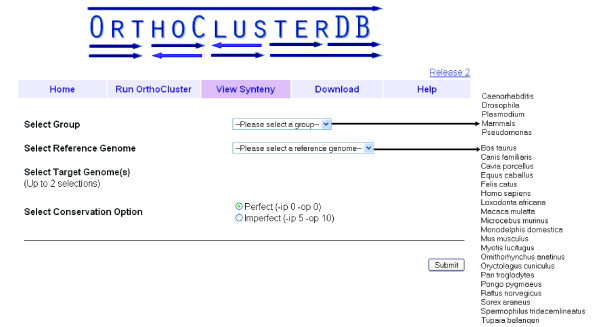
**Web interface of *View Synteny *showing currently available groups of genomes for selection**.

Genomes of some species are of general interest. To facilitate identification of synteny blocks between these genomes, we have created the *View Synteny *page, where users can select predefined genomes of interest for synteny identification and examination. In the current release (Release 2), five groups of genomes are available (Figure 5): *Pseudomonas *(14 genomes), *Plasmodium *(6 genomes), *Caenorhabditis *(2 genomes), *Drosophila *(12 genomes), and Mammals (20 genomes). For these groups, genome files were preloaded and formatted on the web server. The correspondence files for running OrthoCluster are generated on the fly on the web server by parsing precomputed InParanoid [15] results (for two genomes) or by running MultiParanoid [16] (for multiple genomes).

To start a job in the *View Synteny *page, users first select a group of interest and then a reference genome within this group. Once a reference genome is selected, up to two target genomes can be chosen from the same group. Users may choose to identify perfect (no mismatches allowed) or imperfect (containing 5% in-map mismatches, 10% out-map mismatches) synteny blocks. The result page format is the same as that of the *Run OrthoCluster *page.

There are three major differences between the *View Synteny *page and the *Run OrthoCluster *page. First, in the *View Synteny *page, users do not need to prepare input files, making it easier for the user to get quick results for the species of interest. Second, in the *View Synteny *page, genes in the genome browser are linked to their corresponding gene pages in public databases, such as WormBase [17] for the *Caenorhabditis *group, FlyBase [18] for the *Drosophila *group, or Ensembl [19] for the Mammals group. This makes it easy for following up individual genes within synteny blocks in more detail. Third, results from all jobs submitted via the *View Synteny *page are stored permanently in the database (MySQL) of the web server so that results will be immediately returned the next time users try to identify synteny blocks among the same genomes.

### Download

The *Download *page makes available the datasets used by OrthoClusterDB to generate the precomputed results, including the genome annotation files and the pair-wise correspondence files. Genome annotation files were generated based on the GFF (General Feature Format) files obtained from the corresponding model organism databases or, in case of the *Pseudomonas *genomes, from NCBI. Pair-wise correspondence files were generated using Inparanoid [15] with default settings. Also, the standalone version of OrthoCluster for Linux, MacOS, and Windows platforms can be downloaded, allowing users to run OrthoCluster locally on their own computers.

### Computational Platform

OrthoClusterDB is currently supported by a Dual Quad Core Xeon machine that has 8G RAM. The processing time for jobs submitted via *Run OrthoCluster *and *View Synteny *page depends on the number of genes contained in input genomes and the number of orthologous relationships defined in the correspondence file. For pair-wise analysis, jobs usually finish within seconds. For multiple-genome analysis with large correspondence files, jobs may take longer. Such jobs usually take up to one minute to finish on the first run. On the second run, jobs finish immediately because previous results are cached on the server. For larger jobs, users are encouraged to download OrthoCluster from the download page and run it locally.

## Conclusion

Accurate and effective identification of synteny bocks provided by OrthoClusterDB will facilitate many comparative genomics analyses, including the identification of functional gene clusters, ortholog assignment, gene model improvement, identification of lineage-specific genome family expansion and contraction, as well as the characterization of various types of genome rearrangement events such as insertions/deletions, inversions, transpositions, and reciprocal translocations [10]. Currently, OrthoClusterDB allows fast access to precomputed synteny blocks for 54 different genomes within 5 groups of species of general interest. Ultimately, OrthoClusterDB will be expanded to include synteny blocks for all sequenced and annotated genomes.

## Availability and requirements

OrthoClusterDB is free for public access. The engine behind OrthoClusterDB, OrthoCluster, is also free and can be downloaded from the OrthoClusterDB website . OrthoCluster is an effective program and can be run on a single processor. Executables are available for MacOS, PC and Linux. Source code is available upon request.

## Authors' contributions

NC and JP conceived the project. MPN, IAV, CF, QC, XZ implemented the programs. MPN, IAV and NC wrote the manuscript, with input from other co-authors. All authors read and approved the final manuscript.
